# A DNA tumor virus globally reprograms host 3D genome architecture to achieve immortal growth

**DOI:** 10.1038/s41467-023-37347-6

**Published:** 2023-03-22

**Authors:** Chong Wang, Xiang Liu, Jun Liang, Yohei Narita, Weiyue Ding, Difei Li, Luyao Zhang, Hongbo Wang, Merrin Man Long Leong, Isabella Hou, Catherine Gerdt, Chang Jiang, Qian Zhong, Zhonghui Tang, Carmy Forney, Leah Kottyan, Matthew T. Weirauch, Benjamin E. Gewurz, Mu-sheng Zeng, Sizun Jiang, Mingxiang Teng, Bo Zhao

**Affiliations:** 1 Division of Infectious Disease, Department of Medicine, Brigham and Women’s Hospital and Harvard Medical School, 181 Longwood Avenue, Boston, MA 02115 USA; 2 Department of Biostatistics and Bioinformatics, H. Lee Moffitt Cancer Center and Research Institute, Tampa, FL 33612 USA; 3 State Key Laboratory of Oncology in South China, Collaborative Innovation Center for Cancer Medicine, Guangdong Key Laboratory of Nasopharyngeal Carcinoma Diagnosis and Therapy, Sun Yat-sen University Cancer Center, Guangzhou, 510060 China; 4 Zhongshan School of Medicine, Sun Yat-sen University, Guangzhou, 510060 China; 5 Center for Autoimmune Genomics and Etiology, Cincinnati Children’s Hospital Medical Center, Cincinnati, OH 45229 USA; 6 Center for Virology and Vaccine Research, Beth Israel Deaconess Medical Center and Harvard Medical School, Boston, MA 02115 USA; 7 Present Address: Department of Diagnostic and Biological Sciences, School of Dentistry, University of Minnesota, Minneapolis, MN 55455 USA; 8 Present Address: Department of Cancer Physiology, H. Lee Moffitt Cancer Center and Research Institute, Tampa, FL 33612 USA

**Keywords:** Epigenetics, Cancer genetics

## Abstract

Epstein-Barr virus (EBV) immortalization of resting B lymphocytes (RBLs) to lymphoblastoid cell lines (LCLs) models human DNA tumor virus oncogenesis. RBL and LCL chromatin interaction maps are compared to identify the spatial and temporal genome architectural changes during EBV B cell transformation. EBV induces global genome reorganization where contact domains frequently merge or subdivide during transformation. Repressed B compartments in RBLs frequently switch to active A compartments in LCLs. LCLs gain 40% new contact domain boundaries. Newly gained LCL boundaries have strong CTCF binding at their borders while in RBLs, the same sites have much less CTCF binding. Some LCL CTCF sites also have EBV nuclear antigen (EBNA) leader protein EBNALP binding. LCLs have more local interactions than RBLs at LCL dependency factors and super-enhancer targets. RNA Pol II HiChIP and FISH of RBL and LCL further validate the Hi-C results. EBNA3A inactivation globally alters LCL genome interactions. EBNA3A inactivation reduces CTCF and RAD21 DNA binding. EBNA3C inactivation rewires the looping at the *CDKN2A/B* and *AICDA* loci. Disruption of a CTCF site at *AICDA* locus increases AICDA expression. These data suggest that EBV controls lymphocyte growth by globally reorganizing host genome architecture to facilitate the expression of key oncogenes.

## Abstract (web summary)

The dynamic and temporal changes of host genome architecture during Epstein-Barr virus (EBV) transformation are not well known. Here the authors transform human primary B lymphocyte into lymphoblastoid cell lines (LCLs) with EBV and show that the host 3D genome is rewired to facilitate expression of key oncogenes.

## Introduction

Human tumor viruses and other microbes cause approximately 20% of human cancers each year^1^. DNA tumor viruses include Epstein-Barr virus EBV, human papilloma viruses (HPV), hepatitis B virus, and many others. These DNA tumor viruses cause a wide variety of different cancers, including B cell lymphomas, cervical cancer, head and neck cancers, and liver cancers, through expression of viral proteins or RNAs. Viral proteins activate or repress host transcription, leading to increased oncogene expression or reduced tumor suppressor expression^2^.

EBV is the first human DNA tumor virus discovered more than 50 years ago^3^ and causes ~200,000 cases of cancers every year^4^. EBV causes Burkitt’s lymphoma, Hodgkin’s lymphoma, post transplantation lymphoproliferative disease (PTLD), AIDS CNS lymphoma, nasopharyngeal carcinoma, and 10% gastric cancers^5^. These EBV-associated cancers express different latent viral transcription programs that include EBV nuclear antigens (EBNAs) and latent membrane proteins (LMPs)^6^.

EBV transforms short-lived primary human resting B lymphocytes (RBLs) into continuously proliferating lymphoblastoid cell lines (LCLs), in vitro^7^. These cells express the same viral latency genes as some EBV-associated cancers, including PTLD and AIDS CNS lymphomas. LCLs are therefore the ideal model system to study the molecular pathogenesis of EBV-associated cancers. 10 EBV encoded proteins are expressed in LCLs. EBNA1, EBNA2, LP, 3A, 3C, and LMP1 are required for EBV transformation^5^. After EBV infection, EBNA2 and EBNALP are the first EBV proteins expressed. EBNA2 is the major EBV transcription factor (TF) that activates the expression of other viral latency genes and many host genes^8–13^. EBNALP strongly co-activates EBNA2, partly through removal of transcription repressors and activation of EP300^14–17^. In addition, EBNA2 can also modulate the host TF DNA binding, such as RBPJ and EBF1, to enable combinatory TF interactions^15,18^. EBNA3A and EBNA3C repress *CDKN2A/B* expression to overcome virus-induced senescence^19–24^. EBNA3C also promotes cell cycle progression^25–27^. LMP1 activates the NF-κB transcriptional program through both the canonical and non-canonical pathways^28–31^.

Genome-wide analyses of EBV TFs using chromatin immune precipitation (ChIP) followed by deep sequencing (ChIP-seq) find EBV TFs and NF-κB subunits mostly bind to enhancer sites^8,16,19,21,31–33^. These enhancers are sometimes >500 kb away from the transcription start site (TSS), suggesting that a portion of viral-mediated chromatin interactions occur through long-range looping interactions^8,10,13,33^. Inactivation of the viral TFs EBNA2, EBNA3A, or EBNA3C can affect the looping at a number of host loci, mainly exemplified by *MYC*, *CDKN2A/B*, and *BCL2L11*^8,10,13^. However, it is not known if EBV infection of B cells causes global genome reorganization. Using POLR2A Chromatin interaction analysis followed by paired-end tag sequencing (ChIA-PET), we previously linked EBV enhancers to their direct target genes^13^, building the first virus-host 3D genome organization map. Deletion of MYC EBV enhancer sites by clustered regularly interspaced short palindromic repeats (CRISPR) lead to reduced MYC expression, showing the essential nature of many of these regulatory elements targeted by EBV^13^. An alternative silencing method using CRISPR interference (CRISPRi) also downregulated the expression of EBNALP enhancer targets^17^. Previous work characterizing EBV encoded TFs and LMP1-activated NF-κB subunits also show that they assemble EBV super-enhancers (ESEs)^34^. ESEs are also co-occupied by many host TFs and have extraordinary broad and high ChIP-seq signals for active enhancer marks, including the histone modification H3K27ac. ESEs are more sensitive to perturbation than typical enhancers, through both genetic and chemical means^34^. Combinatory analysis of ChIA-PET and CRISPR screen data in EBV-transformed LCLs also shows that ESEs control host genes that are important for LCL growth and survival^13,35^.

To accommodate the small size of the nucleus, the host genome is packaged in extremely complex, yet ordered patterns^36^. Host DNA is packaged in a way that remote enhancers and their direct target genes can communicate rapidly and efficiently, looping out many kilobases of DNA between them^37^. 3D genome interactions can be assessed using chromatin conformation capture followed by deep sequencing (Hi-C), and subsequently identifying the interaction frequencies between genomic loci^36,38–41^. Initial Hi-C studies indicate that the human genome is partitioned into A and B compartments. Genes within A compartment are frequently actively transcribed whereas genes within B compartment are generally repressed^40,42^. DNA tumor virus such as hepatitis B virus can preferentially position its genome at the active host chromatin, likely to be in the A compartments^43^. We investigated the effect of EBV infection on host genome organization to determine if EBV infection causes global genome architectural changes.

In this manuscript, we seek to unravel the effects of EBV, and its viral TFs, on the host genome organization during infection and proliferation, in both space (3D) and time (4D). We generate RBL and LCL Hi-C maps, perform integrative analysis to compile the 4D nucleome of EBV infection of B cells^39^. We validate the Hi-C findings using POLR2A HiChIP in an EBV transformation time course experiment. We next extend our 4D studies by testing if specific EBV protein contributed to global host genome reorganization, using H3K27ac HiChIP in LCLs conditional for EBNA3A expression. In addition, we uncover a role for EBNA3C as a modulator of host genome organization. This represents a comprehensive study into how a DNA tumor virus rewires the host genome during infection, through viral transcription factors, to achieve immortal growth.

## Results

### EBV infection globally changes the host cell 3D genome organization

LCL 3D genome organization is well studied. GM12878 LCL Hi-C, ChIA-PET, and HiChIP data all documented the high-resolution 3D genome architecture of these cells^41,44,45^. However, little is known about the human RBL genome organization and how does it differ from LCL. To understand the dynamic and temporal changes in 3D genome organization between RBL and LCL, we generated Hi-C maps of healthy donor RBL and LCL from the same donor. Primary human B lymphocytes were purified through negative selection. LCLs were generated from these cells 4 weeks after EBV infection. RBLs and LCLs were crosslinked and DNA was cut by HindIII. The DNA ends were filled in with biotinylated dCTP and other nucleotides and ligated. After reverse crosslinking, purified DNA was sonicated. Streptavidin beads captured the ligation products. Purified DNA was paired-end sequenced to generate the Hi-C maps.

**Fig. 1:**
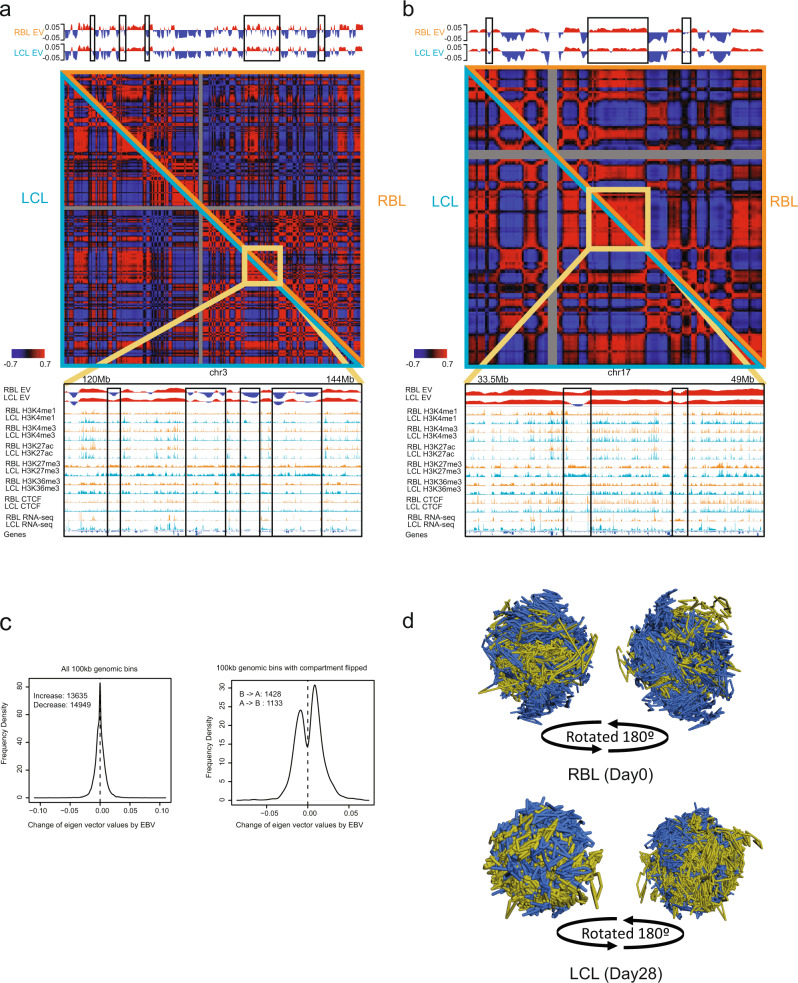
RBL and LCL Hi-C interaction maps. Hi-C correlation maps of resting B lymphocytes (RBL, orange triangle) compared to LCLs (LCL, teal triangle). Pearson correlation (red as high; blue as low) based on normalized interaction frequencies at 500 kb resolution are on the left. Eigenvector values for calling A (red) and B compartments (blue) are shown on top, while the ChIP-seq tracks of histone modifications, CTCF, and RNA-seq in RBLs and LCLs are shown at the bottom for the regions outlined in yellow. **a** Chromosome 3, 120 Mb to 144 Mb. **b** Chromosome 17, 33.5 Mb to 49 Mb. Benjamini-Hochberg adjusted p-values are 9.3e-57 and 1.5e-22 for the highlighted chr3 and chr17 regions, respectively, based on differential compartment analysis of 100 kb genomic regions using tool dcHiC^96^. **c**. Frequency of eigenvector differences for 100 kb genomic bins that have difference signs of eigenvector values between RBL and LCL (left). Frequency of eigenvector differences for all genome-wide 100 kb bins (right). Here, positive difference/change indicates increased eigenvector by EBV infection while negative difference represents decreasing. Numbers in the figure legends indicate the total number of 100 kb bins in the displayed group. **d** 3D genome structure inference of RBL and LCL.

Incorporating Hi-C data and H3K4me3 ChIP-seq, the genome can be divided into transcriptionally active A (red) and transcriptionally repressed B (blue) compartments (Fig. 1a, b, top panels)^40^. Hi-C contact frequency matrices of 100 kb chromosome bins were converted into eigenvectors for both RBL and LCL through eigenvector decomposition^46,47^. The signs of eigenvector values were determined based on Pearson correlation between the eigenvector and H3K4me3 signals. Positive eigenvector represents active chromatin (A compartment). Negative eigenvector represents repressed chromatin (B compartment). A and B compartment switch were evident comparing RBL with LCL (Fig. 1a, b top and c). Similar numbers of compartment flipping events were seen (Fig. 1c, left), indicating global changes with EBV infection. More compartments were flipped from B to A than A to B (Fig. 1c, right). However, the events of deactivation were much weaker compared to the activation, as the range of eigen vector increase was larger than decrease (Fig. 1c, right). Further, genome-wide scanning of 100 kb bins indicated a global increase of eigenvector values (Fig. 1c, right), suggesting chromatin activation happened globally.

Enhancer interactions with specific enhancers or promoters mostly occur within the same higher order genome organization unit, known as contact domains^41,48^. Genomic regions have much higher contact frequencies within the contact domains, than between contact domains. To investigate this in the context of viral infection, Pearson correlation of RBL and LCL contact matrixes were compared at the chromosome level. The comparison between RBL and LCL identified changes in contact domains within the same chromosomes. Small contact domains in RBLs merged into a big contact domain as shown at chromosome 3, between ~120 and 144 Mb (Fig. 1a, yellow box). Within this region, many B compartments in RBLs were converted to A compartments in LCLs, representing a chromosome with abundant B to A conversion (Fig. 1a). Conversely, a large RBL contact domain was converted into three small contact domains in LCLs at chromosome 17 between ~33.5 and 49 MB (Fig. 1b, yellow box). Even though this region was defined as A compartment, junctions at LCL contact domains had lower Eigenvector values than RBLs (Fig. 1b). 3D structural changes also correlated with chromatin status changes along the genome (Fig. 1a, b). For example, active histone marks H3K4me1 signals were significantly higher in regions shifted from B compartment to A compartment at chromosome 3 (Supplementary Fig. 1a) while H3K4me1 signals were significantly lower at chromosome 17 (Supplementary Fig. 1b).

The 3D genome illustrations inferred from the Hi-C data using miniMDS package based on a multidimensional scaling (MDS) method at 10 kb resolution were compared between RBL and LCL (Fig. 1d)^49^. In RBL, A and B compartments tend to be more evenly distributed at the nuclear periphery. In contrast, LCL B compartments tend to congregate on the nuclear periphery (Fig. 1d).

### EBV infection causes dramatic changes in contact domain boundaries

**Fig. 2:**
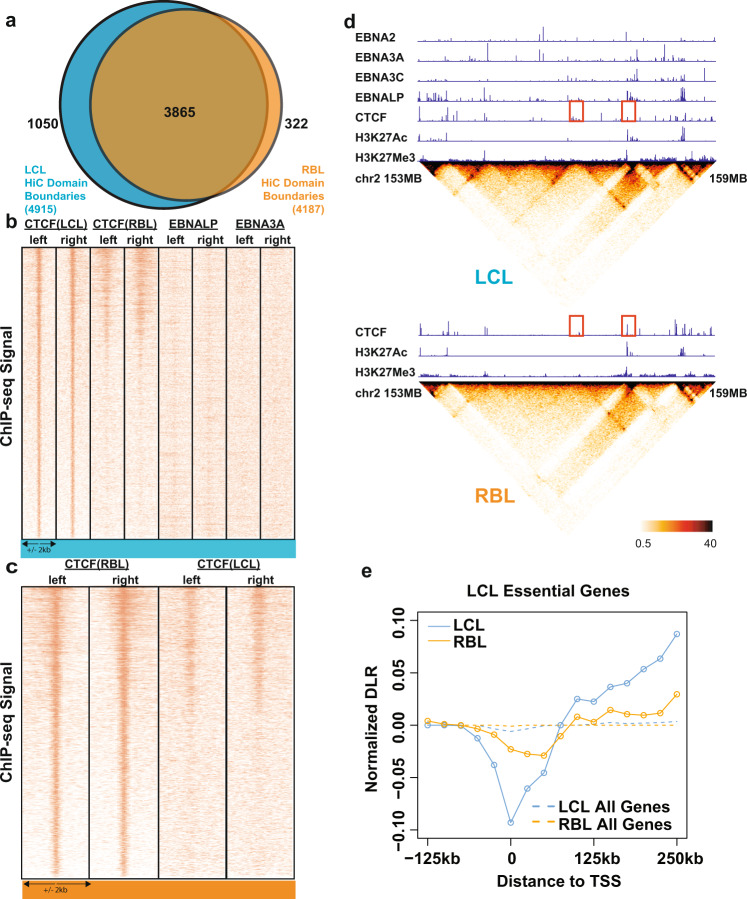
Global changes in contact domain boundaries in RBLs and LCLs. **a** Venn diagram representing the contact domain boundaries from Hi-C in RBLs and LCLs. **b** ChIP-seq signals for CTCF in LCLs and RBLs, EBNALP and EBNA3A were plotted along the edges of LCL unique boundaries (centered on CTCF, +/− 2 kb). Left and right indicate the relative edge positions of the contact domain boundaries. **c**. ChIP-seq signals for CTCF in RBLs and LCLs plotted along the edges of RBL unique boundaries (centered on CTCF, +/−2kb). **d** Representative contact domain changes due to EBV transformation at chromosome 2, 153 Mb – 159 Mb. Top: ChIP-seq tracks and contact frequencies of interactions in LCLs. Bottom: ChIP-seq tracks and contact frequencies of interactions in RBLs. Highlighted in red squares are CTCF profiles that changed at contact domain boundaries between the two conditions. Heatmaps are colored based on normalized interaction frequencies at 25 kb resolution shown at the bottom. **e** Cumulative distal to local interaction (DLR) values at 25 kb resolution near TSSs of genes that are essential for LCL growth and survival (solid lines) and all annotated genes (dotted lines) in both RBLs (orange) and LCLs (teal).

To further illustrate the mechanism through which EBV contributes to 3D genome structure changes, we focused on contact domain boundaries that reside between contact domains. We calculated contact domain boundaries at 25 kb bin resolution with +/− 1 bin flexibility^48^. RBLs had 4187 contact domain boundaries while LCLs had 4915 contact domain boundaries (Fig. 2a). During EBV-mediated B cell transformation, over ~8% of RBL contact domain boundaries were eliminated, while LCLs gained ~21% new contact domain boundaries (Fig. 2a). These data further support a dramatic and global 3D genome reorganization during EBV mediated growth transformation.

CTCF is a key player in contact domain insulation and maintenance of global genome structure^50^. CTCF forms insulators at the boundaries between neighboring chromatin domains, often with opposite transcription activity^51^. To further characterize the properties of contact domain boundaries lost or gained during transformation, ENCODE RBL and LCL CTCF ChIP-seq data were re-evaluated. CTCF signals around CTCF sites and their neighboring +/− 2 kb regions at the edges of contact domain boundaries were compared. For domain boundaries only present in LCL, CTCF signals were evident at the edges of the boundaries in LCLs. At the same sites in RBLs, many had no CTCF signals (Fig. 2b). Interestingly, a portion of LCL gained boundaries were also bound by EBNALP, as EBNALP is known to co-localize with looping factors in LCLs^16^. No evident EBNA3A binding was found at the same boundary sites (Fig. 2b). For domain boundaries only present in RBLs, CTCF signals were evident at the boundary edges in RBLs while at the same sites in LCLs, CTCF signals were present only at ~50% of the sites (Fig. 2c).

As an example, we focused on a new contact domain boundary formed in LCLs at a 6 Mb region on chromosome 2 (Fig. 2d). A cluster of newly formed CTCF sites were evident at the LCL unique boundary (Fig. 2d, top left red square). At the same position in RBLs, weaker CTCF peaks were present inside a contact domain (Fig. 2d, bottom left red square). A prominent CTCF peak at the edge of RBL contact domain (Fig. 2d, bottom right red square) became greatly weakened in LCLs and was confined within the newly formed LCL contact domain (Fig. 2d, top right red square). CTCF binding sites on both sides of the altered contact domains remained mostly unchanged.

To determine if the genome reorganization occurred at genes essential for LCL growth and survival identified by a genome-wide CRISPR screen^32^, distal to local interaction ratio (DLR) was evaluated at these genes^35,42^. Local interactions are defined as interactions between a genomic region and other genomic regions within a 3 Mb window. Distal interactions are defined as those between the same genomic region and other genomic regions outside the 3 Mb window. A negative DLR indicated more local interaction compared to distal interaction and enrichment in local looping. Cumulative DLR values at 25 kb resolution near TSSs were determined for LCL dependency factors in both RBLs and LCLs (Fig. 2e, solid lines). Baseline DLR values, across all annotated genes were also plotted (Fig. 2e, dotted lines). LCLs had more local interactions at TSS for genes essential for LCL, while the rest of the genes had similar local and distal interactions. Comparing with LCLs, RBLs had less local interactions at TSS (Fig. 2e). The regions upstream of TSS had more distal interactions in LCLs than RBLs (Fig. 2e). These data indicated that the genomic loci that harbor genes essential for LCL growth and survival undergo global genome reorganization during EBV transformation to ensure the optimal expression of these LCL dependency factors. The dip in DLR for RBLs at essential LCL genes, although smaller than that in LCLs, represents a pre-existing local interactions present at these genes (Fig. 2e, orange and teal solid lines). Together, these data suggested that these essential gene promoters are “primed” in B cells for a growth and proliferation program (such as in the event of B cell activation by T cells), which is usurped by the oncogenic EBV during transformation.

99% of LCL ubique domain boundaries are enriched with CTCF binding in LCL, but only ~47.5% of them are also bound by CTCF in RBL. Similarly, ~98.3% of RBL unique domain boundaries are enriched with CTCF binding in RBL, but only ~22.2% are enriched with CTCF in LCL. In addition, about 20.3% of LCL CTCF bound domain boundaries are enriched with EBNALP binding in LCL, among which 76% are sites also bound by CTCF in RBL.

### Genome reorganization at ESEs

**Fig. 3:**
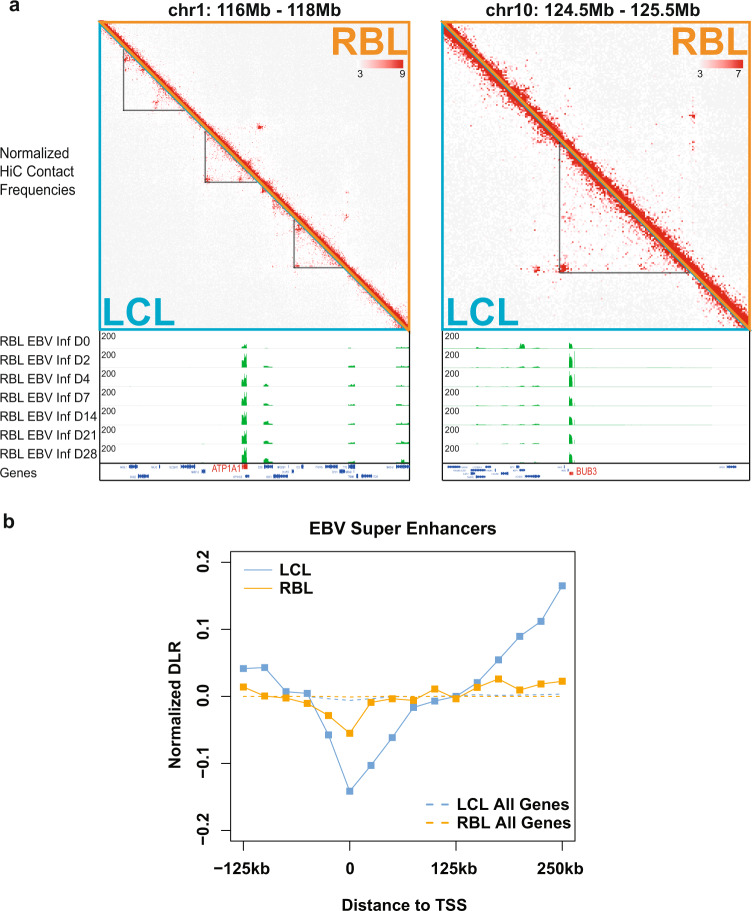
Global changes in contact domain at ESEs and their target genes. **a** Normalized Hi-C contact frequencies for RBLs (orange triangle) and LCLs (teal triangle) at 2 representative loci are shown on top. Left: Chromosome 1, 116 Mb to 118 Mb. Right: Chromosome 10, 124.5 Mb to 125.5 Mb. Visible changes in LCL domains are traced out in black, and RNA-seq tracks for RBL after EBV infection are shown at the bottom. Heatmaps are colored based on normalized interaction frequencies at 10 kb resolution. **b** Cumulative DLR values at 25 kb resolution near TSSs of genes regulated by EBV Super Enhancers (ESEs, solid lines) and all annotated genes (dotted lines) in both RBLs (orange) and LCLs (teal).

Since ESEs are important for LCLs growth^13,34^, we examined the genome reorganization around ESEs during EBV transformation. Increased genomic interactions in LCLs were evident around ESEs comparing with RBLs (Fig. 3a, b).

Two ESEs located at the *ATP1A1-CD58* loci, spanning a ~ 300 kb genomic region (Fig. 3a, left). ATP1A1 is a Na^+^/K^+^ pump and is important for tumor metastasis^52^. CD58 signaling can cause isotype switching and cytokine production^53,54^. Expression levels of ATP1A1 and CD58(LFA3) correlated with poor prognosis in liver cancers^55^. During EBV transformation of RBL into LCLs, ATP1A1 and CD58 expression greatly increased by RNA-seq analyses (Fig. 3a, left). In LCLs, these genes and their neighboring regions had higher interaction frequencies in Hi-C. In contrast, RBLs had much less interactions within the loci (Fig. 3a, left). These changes were accompanied by the dramatic increase in gene expression at these loci.

At the *BUB3* locus, one ESE is linked to *BUB3* (Fig. 3a, right). BUB3 is a spindle checkpoint protein that is important for cell cycle progression. BUB3 is frequently implicated in cancer for causing genome instability^56^. In LCLs. The genomic regions within this locus had high genomic interaction frequencies. However, much less genome interactions were found within this locus in RBLs (Fig. 3a, right). The chromatin reorganization at the locus was accompanied by increased BUB3 expression (Fig. 3a, right).

To determine the global effect of EBV infection on genome interactions at ESE associated genes^13^, DLRs were also determined at the genes linked to ESEs by POLR2A ChIA-PE in RBLs and LCLs. In LCLs, these TSSs had more local interactions than distal interactions. In comparison, RBLs had slightly less local interaction between TSSs and their immediate neighboring regions (Fig. 3b).

**Fig. 4:**
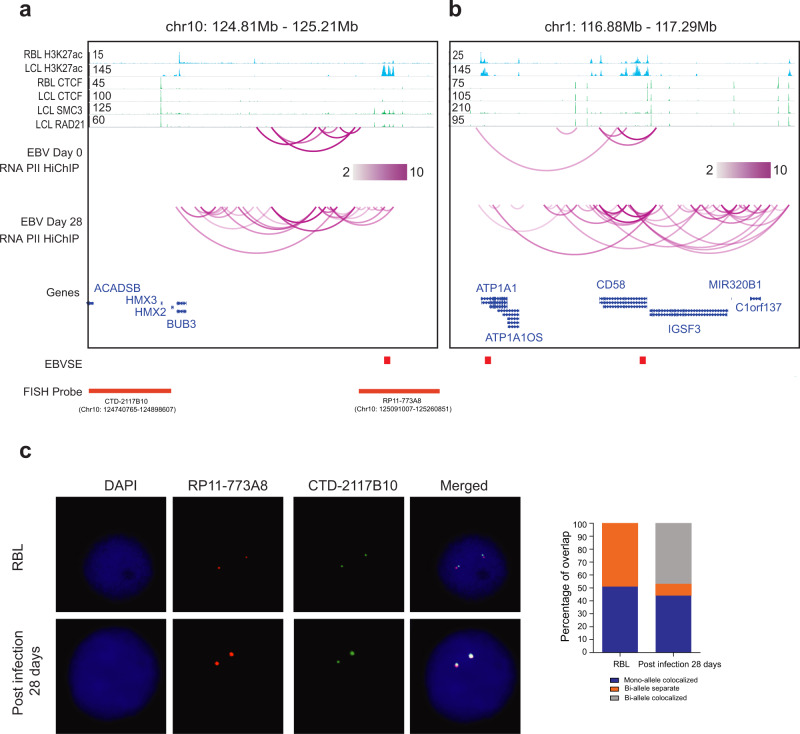
ESEs and their direct target interactions in RBL to LCL time course. **a** RBLs (day 0) were transformed into LCLs (day 28) in a time course experiment in replicates. ESE linkages to their direct genes were evaluated by POLR2A HiChIP. Each magenta line represents a genomic interaction. HiChIP links with filter threshold >2 are shown. HiChIP loops in RBLs and LCLs at a. ATP1A1 and CD58, **b** BUB3. RBL and LCL H3K27ac, CTCF and LCL RAD21, SMC3 ChIP-seq tracks are also shown on the top. **c** RBLs and LCLs 28 days post infection were fixed and hybridized with labeled BACmids. The nuclei were stained with DAPI. The BAC probes used are indicated in panel **b**. More than 50 nuclei were scored for each cell type and tabulated on the right. Pearson’s Chi-square test *P* < 3.69-e-9. Source data are provided as a supplementary Source Data file.

To further validate the genome organizational changes found comparing RBL and LCL Hi-Cs, POLR2A HiChIP was used to evaluate differential looping patterns during RBLs to LCLs transition in an EBV transformation time course^45^. Purified RBLs were infected with wild-type EBV and grown in culture media for 4 weeks to establish LCLs. RBL and LCL cells were crosslinked and DNA was cut by MboI. DNA ends were filled with biotinylated dATP and other nucleotides and then ligated in situ. The ligated DNA linked by POLR2A were enriched by ChIP. Reverse crosslinked DNA was selected with avidin beads and paired-end deep sequenced. In LCLs, abundant interactions were evident between ESEs and their direct target genes, or ESE first interacted with neighboring regions and then looped to direct target genes (Fig. 4a, b). Frequent interactions were also evident between ESE. In contrast, little interactions were observed between ESEs and their direct targets in RBLs (Fig. 4a, b). These data further illustrated the spatial and temporal genome reorganization during EBV transformation.

To estimate the frequency of interactions between ESE and *BUB3* in RBL and LCL population, fluorescence in situ hybridization (FISH) was used. RBLs and 4 week LCLs from the same donor were hybridized with BACmids targeting *BUB3* and ESE. In RBLs, ~50% of the cells had monoallele colocalization and ~50% biallele non-colocaliztion. In LCLs, ~50% of the cells had biallele colocalization and ~50% monoallele colocalization (Fig. 4c). These data further support the increased interactions between ESE and BUB3 in LCLs identified by Hi-C and HiChIP.

### EBNA3A changes global 3D genome reorganization

EBNA3A is essential for EBV transformation of RBLs into LCLs. Recombinant EBV deleted for EBNA3A fails to immortalize RBLs^57^. LCLs expressing a conditional EBNA3A fused to modified estrogen hormone binding domain (EBNA3AHT) grow normally in the presence of 4-hydroxytamoxifen (4HT). In the absence of 4HT, LCLs enter growth arrest with increased p14^ARF^ and p16 ^INK4A^ expression^20,22,58^. EBNA3A is tethered to DNA through interactions with host TFs^21,33^ and regulates host gene expression^59–62^. EBNA3A can bind to RBPJ, a Notch pathway protein, and USP46/USP12 or CtBP^63–65^. EBNA3A is also involved in long range enhancer-promoter interaction^13^. However, it is not known if EBNA3A can affect the global host 3D genome organization.

To evaluate the genome-wide effect of EBNA3A on host 3D genome organization, H3K27ac enriched enhancer interactions with their direct targets were analyzed using HiChIP assay. Conditional EBNA3AHT LCLs were grown under permissive or non-permissive conditions for 14 days followed by H3K27ac HiChIP.

**Fig. 5:**
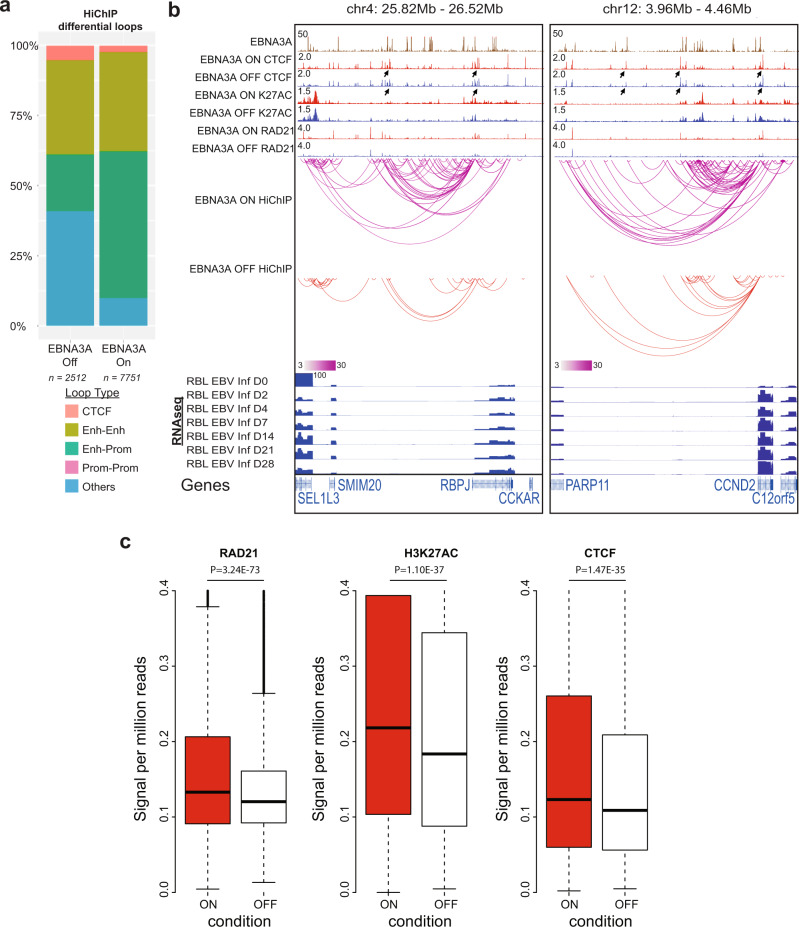
EBNA3A induced global changes in chromatin interactions. **a** H3K27ac HiChIP experiments were used to identify chromatin loops in EBNA3A conditional LCLs grown under permissive (EBNA3A On) or non-permissive (EBNA3A Off) conditions. EBNA3A inactivation (EBNA3A Off) resulted in a marked decrease in the number of loops formed, decrease in enhancer-promoter loops as well as increases in CTCF loops and other unannotated loops. All HiChIP loops have FDR < 0.01 and *p*-value <0.05 for differential enrichment between EBNA3A On and Off conditions. **b**-left Representative genomic tracks on chromosome 4, around the RBPJ gene locus. EBNA3A inactivation resulted in decreased chromatin loops around RBPJ, particularly at sites with EBNA3A and chromatin looping factors (CTCF, SMC3, and Rad21) present. RNA-seq tracks tracing gene expression throughout the EBV infection of B cells show an increase in RBPJ transcripts. HiChIP links with filter threshold >3 are shown. Black arrows indicate different CTCF binding pattern in RBLs and LCLs. **b**-right Representative genomic tracks on chromosome 12, around CCND2. EBNA3A inactivation resulted in decreased chromatin loops throughout the locus. HiChIP links with filter threshold >3 are shown. EBV infection of B cells induced increase in gene transcription is shown at the bottom. **c** CTCF and H3K27ac Cut & Run and RAD21 ChIP-seq signals at the genomic loci that lost looping upon EBNA3A inactivation. Boxplot plots: center value is the medium; upper and lower bounds of boxes are upper and lower quartile, respectively; whiskers extend by 1.5*(upper quartile - lower quartile). *P* values were calculated using the Wilcoxon rank sum test.

In LCLs grown under permissive condition for EBNA3A expression, HiChIP identified 7429 significant H3K27ac loops between enhancer-enhancer, enhancer-promoter, CTCF-CTCF, promoter-promoter, or enhancer-unannotated sites (FDR < 0.01 and *p* < 0.05). In LCLs grown under non-permissive conditions, HiChIP identified much less loops, with 2508 significant H3K27ac loops between enhancer-enhancer, enhancer-promoter, CTCF site, enhancer-unannotated site, or promoter-promoter were identified (*p* < 0.05). In the EBNA3A on condition, 2.4% of the loops were between CTCF sites, 35.5% of the loops were enhancer-enhancer, 52% of the loops were enhancer-promoter, and 10% of the loops were enhancer-unannotated site. EBNA3A inactivation slightly increased the fractions of loops between CTCF site (5%) and greatly increased enhancer-unannotated site (40.7%), but greatly decreased enhancer-promoter loops (20.5%), while enhancer-enhancer interactions remained similar (33.5%) (Fig. 5a).

EBNA3A inactivation caused significant reductions in enhancer-promoter loops. Many of these loops linked to genes essential for LCL growth and survival, these include *RBPJ* and *CCND2* (Fig. 5b). EBNA3A inactivation greatly reduced the enhancer-promoter loops regulating RBPJ. RBPJ is essential for LCL growth and survival. EBNA2, 3A, 3B, and 3C all bind to RBPJ. EBNA2, 3A, and 3C with mutations in their RBPJ binding sites cannot support LCL growth. EBNA3A and 3C can block RBPJ DNA binding in vitro. EBNA3A inactivation also decreased RAD21, CTCF, and H3K27ac signals at these loci (Fig. 5b).

Some genes gained loops upon EBNA3A inactivation, these included *CCR2* and *CCR5* (Supplementary Fig. 2a–d). ChIP-seq signal enrichment analysis at differentially looped anchors identified a number of host TFs that were either enriched or depleted, including ETS1, MEF2C, and BATF (Supplementary Fig. 3).

H3K27ac Cut & Run was used to evaluate the effect of EBNA3A inactivation on active enhancer mark. EBNA3A inactivation significantly reduced the H3K27ac signals at the sites that lost loopings after EBNA3A inactivation (Wilcoxon Rank Test, *P* = 1.10E−37, Fig. 5c). The effect of EBNA3A inactivation on CTCF DNA binding was also evaluated by CTCF Cut & Run. The CTCF signals at the sites lost looping after EBNA3A inactivation also were significantly reduced (Wilcoxon Rank Test, *P* = 1.47E−35, Fig. 5c). Cohesin family members RAD21, SMC1A, and SMC3 are essential for host genome organization. RAD21, SMC1, and SMC3 form ring like structure and wrap around DNA, allowing chromatin loops to extrude through the ring, therefore bringing distant enhancer-promoter into close proximity with each other^66^. As part of the loop extrusion model, cohesin rings are locked in place by strong CTCF homodimerization as part of the way chromatin intearctions are regulated. RAD21 ChIP-seq was used to evlaueate the effect of EBNA3A inactivation on RAD21 DNA binding. EBNA3A inactivation also significantly reduced RAD21 DNA bind at the genomic loci that lost looping upon EBNA3A inactivation (Wilcoxon Rank Test, P = 3.24E−73, Fig. 5c).

### EBNA3A inactivation alters ESE-target gene connections

**Fig. 6:**
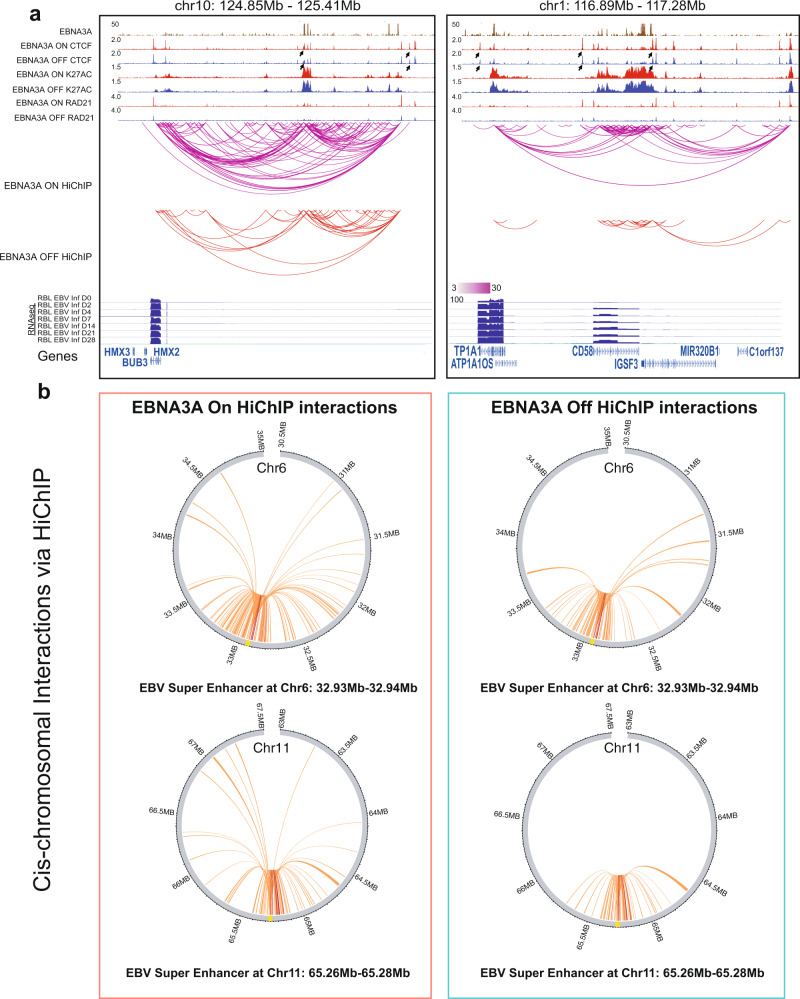
EBNA3A inactivation reduces ESEs loopings. **a** Inactivation of EBNA3A resulted in significantly less interactions between ESEs at chromosome 10 (BUB3, left) and chromosome 1 (ATP1A1 and CD58, right) and their direct target genes. HiChIP links with filter threshold >3 are shown. Black arrows indicate different CTCF binding pattern in RBLs and LCLs. **b** Cis-chromosomal loops were visualized using Circos plots, originating from two ESEs. The cis-chromosomal loops in EBNA3A On (left) are decreased upon EBNA3A inactivation (right).

EBNA3A inactivation reduced *MYC* ESEs looping to MYC TSS by 3C-qPCR^13^. H3K27ac HiChIPs were used to evaluate the genome-wide effect of EBNA3A inactivation on ESE loopings. EBNA3A inactivation significant reduced H3K27ac loops at the *ATP1A1-CD58* loci (Fig. 6a). EBNA3A binding sites were evident at the ESEs together with high H3K27ac signals. In the presence of EBNA3A, abundant interactions looped between ESE located near *CD58* and ESE near *ATP1A1*. EBNA3A inactivation greatly reduced the looping between the two ESEs. The only remaining loopings were limited around *CD58* in EBNA3A off condition (Fig. 6a). Multiple CTCF sites and RAD21 sites were evident at the loci. EBNA3A inactivation also greatly reduced H3K27ac interactions between ESEs and *BUB3* (Fig. 6a). An ESE was located >200 kb downstream from *BUB3* TSS. Strong EBNA3A peaks were evident at the ESE while no EBNA3A peak was near *BUB3*. We also observed an increase of the loopings at these two loci in the EBV infection time-course HiChIP experiment (Fig. 4a, b).

Some ESEs link to multiple targets genes^13^. To understand the effects of EBNA3A on ESE looping, we focused on two ESEs with the greatest number of cis-interactions. EBNA3A inactivation reduced H3K27ac looping from ESEs to multiple direct target genes (Fig. 6b).

### EBNA3C inactivation reorganizes the CDKN2A/B locus

**Fig. 7:**
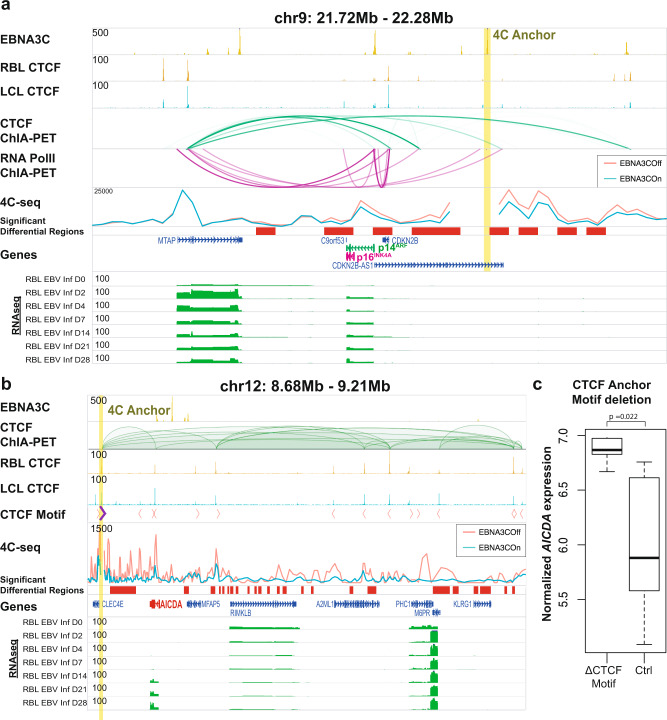
EBNA3C alters loopings at CDKN2A/B and AICDA. **a** Chromatin interactions at the CDKN2A/B locus is shown, with the 4C-seq anchor shown at a distal EBNA3C peak residing upstream of the p14^ARF^ and p16^INK4A^ promoters. Significant differential regions (*p* < 0.05) are shown in red bars, which overlap with a number of regions previously identified by CTCF and POL2RA ChIA-PET. EBNA3C inactivation (EBNA3C Off) resulted in increased chromatin interactions of regions upstream of p14^ARF^ and p16^INK4A^ with its promoters, indicating putative regulatory regions that are suppressed by EBNA3C to downregulate p14^ARF^ and p16^INK4A^. **b** Chromatin interactions at the AICDA locus is shown, with the 4C-seq anchor at a key CTCF site involved in multiple interactions at this locus. Significant differential regions (*p* < 0.05) are shown in red bars, which overlap with the majority of CTCF peaks and motifs indicated. The directionality of CTCF motifs are also indicated. The presence of EBNA3C (EBNA3C On, teal) resulted in a significant suppression of local CTCF interactions, which is marked by an increase in *AICDA* and *M6PR* expression. **c** The key CTCF motif, marked in purple in **b**, was deleted via CRISPR-Cas9. Single cell clones of LCLs harboring these deletions were grown out and verified for successful or unsuccessful motif deletion. The gene expression after successful CTCF motif deletion was significantly higher than wildtype, implicating the role of CTCF interactions, when disrupted by EBNA3C, in upregulating *AICDA* expression. Boxplot plots: center value is the medium; upper and lower bounds of boxes are upper and lower quartile, respectively; whiskers extend by 1.5*(upper quartile - lower quartile). Wilcoxon test, two sided, *p* = 0.022. Source data are provided as a supplementary Source Data file.

EBNA3C can regulate long-range looping at several genes essential for LCL growth, including *MYC* and *CDKN2A/B*^13^. EBNA3C repression of *CDKN2A/B* gene expression is essential for LCL to escape senescence^20^. EBNA3C decreases the interactions between *p16*^*INK4A*^*, p14*^*ARF*^, and *p15*^*INK4B*^ promoters^13^. EBNA3C binds to the *p14*^*ARF*^ promoter and recruits transcription repressor SIN3A to this locus^19^. To further determine the effect of EBNA3C on the local chromatin interactions at the *CDKN2A/N* locus, circular chromatin conformation capture followed by deep sequencing (4C-seq) was performed in EBNA3C conditional LCLs. Conditional EBNA3C LCLs grown under permissive or nonpermissive conditions were crosslinked and lyzed. DNA was first cut with HindIII and ligated. DNA was purified after reverse crosslinking and cut again with Dpn II. The DNA fragments were circularized by ligation and inverse PCR was done to amplify the DNA ligated to viewpoint followed by deep sequencing. The viewpoint (Fig. 7a, anchor as indicated by yellow vertical bar) was determined through an assessment of suitable restriction enzyme fragments which encompassed a key distal EBNA3C peak. We found that under EBNA3C permissive conditions, the distal EBNA3C peak interacted with multiple genomic regions across this locus (Fig. 6a, teal lines, EBNA3C On). Previous CTCF and POL2RA ChIA-PET interactions in LCLs corroborated the 4C-seq interactions (Fig. 6a, green and purple loops). When EBNA3C was inactivated, the interaction frequencies increased in the regions across the locus, including the senescence genes *p15*^*INK4B*^, *p14*^*ARF*^, and *p16*^*INK4A*^ (Fig. 7a, orange lines and red bars, *p* < 0.05). These results suggest to a model in which EBNA3C represses local chromatin interactions, particularly enhancer-promoter interactions, at the CDKN2A/B locus to repress genes activated as part of the cellular senescence during EBV infection and LCL establishment.

### Loss of a CTCF site downstream of *AICDA* up-regulates AICDA expression

*AICDA* encodes for a vital B cell protein, AID, which is important in regulating class switch recombination and somatic hypermutation^67^. AID-induced chromatin breaks at the MYC locus are required for chromosome translocations that result in the *MYC* and immunoglobulin enhancer fusions prevalent in Burkitt’s lymphomas^68^. Interestingly, EBNA3C up-regulates AICDA expression, which plays a role in increasing global mutational burdens in LCLs^69^. A detailed analysis of the *AICDA* locus identified a key CTCF site downstream of *AICDA*, looped towards multiple sites upstream shown by LCL CTCF ChIA-PET (Fig. 7b, CTCF motif in purple). The directionality of the CTCF motif is a key determinant of its insulator function^44^, and this particular motif was oriented towards the upstream direction, insulating all its local interactions in that directionality (Fig. 7b, green loops). During our analysis, we observed that EBNA3C was bound near a CTCF site at the *AICDA* promoter, which was looped to another CTCF site downstream (Fig. 7b). To understand how EBNA3C controls looping around this locus, we identified a suitable 4C-seq viewpoint that encompasses the key CTCF motif (Fig. 7b, anchor as indicated by yellow vertical bar). Nla III and Csp 6I were used in 4C-seq. Inactivation of EBNA3C resulted in a significant increase in interactions originating from this CTCF anchor, notably with the multiple other CTCF peaks in this locus (Fig. 7b, orange/teal lines and red bars). EBNA3C can therefore reduce CTCF insulator contacts at this locus (*p* < 0.05). With 4C-seq analysis, we speculate that the local chromatin conformation maybe accessible upon EBNA3C activation and condensed upon EBNA3C inactivation.

CRISPR/Cas9 deletions was performed find a causal role of the CTCF motif on *AICDA* expression. To reduce potential off-target effects through long-termed expression of the Cas9 protein and its associated sgRNAs, we nucleofected LCLs with ribonucleoprotein (RNP) complexes of Cas9 and sgRNAs (see Material and Methods). Single cells were cloned out from nucleofected LCLs through serial dilution, and the expression of *AICDA* quantified for clones that successfully underwent deletion (100% of motif deleted), or clones that received RNPs but did not have an observable deletion (0% of motif deleted) (Fig. 7c). Disruption of this CTCF motif resulted in a visible increase in *AICDA* expression (Fig. 7c, *p* < 0.05). Taken together, EBNA3C disrupts local CTCF interactions at the *AICDA* locus to increase the expression of this key B cell protein, thereby increasing the mutational burden of LCLs.

## Discussion

Here we report that the host genome undergoes global reorganization during a prototypical human DNA tumor virus infection and transformation of host cells into cancer-like cells. Defining the nuclear spatial and temporal changes during viral infection is a crucial discovery step towards understanding the molecular pathogenesis of virus infection. Understanding how these changes occur during immortalization will not only provide insight into EBV oncogenesis, but also elucidate transcriptional regulation during normal B cell activation.

Previous incorporation of LCL POLR2A ChIA-PET data with viral and host TF ChIP-seq data allowed the generation of the LCL 3D genome landscape, linking LCL enhancers to their direct target genes^13^. This map highlighted the key components governing the regulation of key oncogene expression, through validation studies using CRISPR deletion of enhancers, or via essential genes identified through a companion CRISPR screen in LCLs^13,35^. However, little is known about the temporal genome organizational changes during EBV infection and subsequent growth transformation, and how these changes drive gene expression. Therefore, it is important to track the 3D genome organizational changes between RBLs and LCLs systematically.

POLR2A ChIA-PET selectively enriches for enhancer-enhancer and enhancer-promoter interactions linked by POLR2A^44^. However, the large number of cell input required for a ChIA-PET experiment presented a technical hurdle to study primary RBLs, and the infection process. The lower cell number input of the Hi-C assay also allows a robust construction of a high resolution LCL 3D genome interaction map, albeit at the cost of a higher depth of sequencing^39^. In this study, we therefore first used Hi-C to define the RBL genome organization map and compared it with LCLs, to delineate the temporal changes following viral infection. We then used a combination of H3K27ac HiChIP and 4C-seq to further determine the contributions of individual EBV oncogenes on host 3D genome organization.

Mouse resting B cells undergo dramatic genome organizational changes when stimulated by LPS and IL4, resulting in vastly increased numbers in contact domains^70^. Similarly, we discovered that human RBLs and LCLs also significantly differed in their chromatin contact domain numbers. Furthermore, we found that in comparison to uninfected RBLs, LCLs have increased local chromatin interactions at ESEs and their associated genes, indicating that EBV is involved in SE assembly to alter host genome organization. LPS and IL4 signaling activate a cascade of transcription factors, such as NF-kB and STAT6; similarly, LCLs have high NF-kB activity and express 4 essential EBNAs. EBNAs can modulate host histone modifying enzymes to alter the epigenetic landscape, which may lead to differential binding of host TFs and looping factors on DNA^17,71,72^. In parallel, EBV infection can also affect global host DNA methylation, which can affect the specific binding of specific TFs, notably CTCF^73–75^. CTCF can function as insulator, blocking the spread of chromatin modifications from one region to the next region^76^, while cohesin subunits form rings to extrude out DNAs between enhancers and their regulated genes. Activated mouse B cells have much higher RAD21 ChIP-seq signals at the induced loops, but only modestly higher CTCF ChIP-seq signals;^70^ further work will be needed to understand how B cell activation can affect the DNA occupancy of these looping factors. We elucidated in this study, that DNA occupancy of looping factors CTCF, SMC1, and RAD21 can be affected by EBNA2, 3A, and 3C in LCLs. Furthermore, EBNALP frequently co-localizes with looping factors, although the EBNALP-specific interactions may be difficult to tease out at the moment, due to the lack of a conditional genetic system^16^. Future work should focus on the detailed molecular mechanism through which EBV alters looping factor DNA binding during infection and transformation.

SEs play critical roles in cell growth and differentiation^77^. Reduced expression of ESE key EBV components (EBNA2, EBNA3A, and EBNA3C) significantly reduced the looping. The assembly of ESE may lead to the formation of new contact domains, as ESEs can interact with multiple downstream target genes through long ranged chromatin interactions. In addition to looping factors studied here, ZNF143 and YY1 are also key factors whose roles are yet to be fully elucidated^78,79^. It has not escaped our notice that previous EBNA ChIP-seq studies identified a significantly enriched of these motifs near EBNA binding sites^8,16,80^. A thorough, global analysis of how EBV usurps the B cell transcription program through host TFs will be paramount to better understand the intricacies of both viral and host processes.

## Methods

### Cell lines and antibodies

The GM12878, EBNA3A-HT, and EBNA3C-HT LCLs were previously described^12,24,58^. All LCLs were grown in RPMI (Gibco) supplemented with 1% L-glutamate, 1% Pen/Strep, and 10% FBS. B958 ZHT, P3HR1 ZHT cells, and virus induction were previously described^81,82^. 1μg anti- CTCF (Abcam Cat: #ab70303) antibody was used for CUT & Run; 1μg anti-H3K27ac (Abcam Cat: #ab4729) antibody was used for CUT & Run; 12μg anti-RAD21 (Abcam Cat: #ab992) antibody was used for ChIP-seq. 8ug Anti-RNA Polymerase II RPB1 (Biolegend Cat: #664906) antibody was used for HiChIP.

### Primary human B cells and LCLs

De-identified blood cells were obtained from the Gulf Coast Regional Blood Center, following institutional guidelines. The Epstein-Barr virus studies described in this paper were approved by the Brigham & Women’s Hospital Institutional Review Board. B cells were purified via negative selection, with RosetteSep Human B Cell Enrichment Cocktail and EasySep Human B Cell Enrichment Kits (StemCell Technologies), according to the manufacturer protocols. LCLs were generated from RBLs infected with B95.8 strain EBV for 28 days.

### Hi-C

Hi-C was done following the Arima-HiC Kit protocol (Arima, A510008). 10 million cells were crosslinked with 2% formaldehyde for 10 min at room temperature. The reaction was quenched using 200 mM glycine for 5 min. Samples were washed in PBS once, then resuspended in 1 ml of cold Hi-C lysis buffer (10 mM Tris-HCL PH 8.0, 10 mM NaCl, 0.2% NP-40,1x Protease inhibitors) and kept on ice for 15 min. Nuclei were spun down at 2500 x g for 5 min and the supernatant were discarded. Pelleted Nuclei were washed once with 1 ml of cold Hi-C lysis buffer then resuspended in 450ul dH2O containing 15ul 10% SDS shaking at 900 rpm for 1 h in 37 °C. 75ul 20% Triton X-100 was added to quench the SDS. The chromatin was digested with 600 units HindIII (New England Biolabs) at 37 °C overnight with shaking. Restriction enzyme was inactivated by incubating 30 min at 65 °C. The single-strand overhangs were filled in with 37.5 ul of 0.4 mM biotin-14-dCTP (Life Technologies) by 10ul of 5U/ul Klenow DNA polymerase (New England Biolabs) for 1 h at 37 °C. The biotinylated DNA was suspended in 6.6 ml ligation mix (5.4 ml dH2O,700 ul 10xligase buffer,375ul 20%Triton X-100, 80ul 10 mg/ml BSA, 50ul 1U/ul T4 DNA ligase). DNA was ligated overnight at 16 °C by slow rotating. Proteins were removed by adding 30ul of 10 mg/ml proteinase K (New England Biolabs) and incubated at 55 °C for 30 min. Crosslinking was reversed with incubation at 65 °C overnight. DNA was purified with Phenol-Chloroform extraction and followed by ethanol precipitation. Biotin dCTP at non-ligated DNA was removed with 5 units T4 DNA polymerase. 5ug of Hi-C DNA pellets were dissolved in 130ul 1xTris buffer and sonicated. Covaris LE220 with parameter (Duty Cycle:15, Cycles/Burst:200, Time:1 min) sheared DNA to 300–700 bp fragments. Hi-C library was prepared using size selection with Ampure XP beads. Biotinylated DNA was recaptured by 100ul Dynabeads MyOne C1 Streptavidin beads (Life Technologies). Sequencing libraries were directly amplified on C1 beads with 8 cycles of PCR using illumine primers and protocol. After PCR, solutions were placed on a magnet and libraries were eluted into new tubes. The libraries were then purified with DNA Clean and Concentrator columns to a volume of 10ul. The sequencing libraries were checked using an Agilent Bioanalyzer 2100 and quantified using a Qubit (Life Technologies). Libraries were sequenced on an Illumina NextSeq 500 with 75 cycles of paired-end reads.

### ChIP-seq

ChIP-seq assays were done as previously described^8^. In brief, 1 × 10^7^ cells were fixed with formaldehyde and lyzed. Cell lysates were sonicated and diluted. The lysates were precleared with protein A beads and the incubated with antibody overnight at 4 °C, rotating. The protein-DNA complexes were captured by protein A beads. After extensive wash, the protein-DNA complexes were eluted and reverse crosslinked. Qiagen PCR purification kits were used to purify the DNA. Libraries were prepared using Illumina DNA library prep kit (E7645S).

### Cut & run

Cut and run was done following the protocol from CUTANA™ ChIC/CUT&RUN Kit (Epicypher, 14-1048). In brief, 0.5 million cells per sample were harvested and washed with PBS once. Nuclei were isolated with nuclear extraction buffer and then captured with activated ConA beads. 1ug antibody targeting protein of interest was added to nuclei solution and incubate at 4°C with shaking overnight. DNA was cleaved with PAG-MNASE and released to solution. DNA was then purified. The library was prepared using Illumina DNA library prep kit (E7645S).

### HiChIP

H3K27ac HiChIPs from LCLs conditional for EBNA3A expression were performed as previously described^45^ using H3K27ac antibody (Abcam). Briefly, 1 × 10^7^ cells for each biological replicate were collected and cross-linked by 1% formaldehyde for 10 min. Chromatin was digested using MboI restriction enzyme (New England Biolabs). DNA ends were filled in with Biotin-14-dATP (Thermo Fisher) and other nucleotides and then ligated. After sonication, sheared chromatin was pre-cleared and 3-fold diluted as described in ChIP method and then incubated with 4ug anti-H3K27ac antibody at 4 °C for overnight. Chromatin-antibody complex was captured by Dynabead Protein-A bead, followed by capture with Streptavidin C-1 bead (Thermo Fisher). Libraries were generated using Tn5 followed by PCR. HiChIP samples were size selected by PAGE purification (300–700 bp). All libraries were sequenced on the Illumina NextSeq 500. Each sample has an average depth of ~20 million reads.

### FISH

RBLs and 4-week LCLs from the same donor were swelled in 0.075 M KCl at room temperature for 20 minutes and fixed with 3:1 Methanol: Acetic Acid. BACmids RP11-773A8 and CTD-2117B10 were labeled with green or orange dUTP (Abbott Molecular). Fixed cells on the slides were hybridized with the labeled BACmid probes. The nuclei were stained by DAPI.

### Hi-C data analysis

Raw reads for LCLs and RBLs were processed with HiC-Pro pipeline first (version v3.1.0)^83^ to obtain putative interactions with default parameters under genome build hg19. Contact maps were generated with bins under different resolutions (5k, 10k, 25 kb, 50k, 100 kb, 500 kb) in order to fit downstream analysis at different scales. Contact maps were normalized with iterative correction and eigenvector decomposition (ICE)^47^ using the version implemented within the HiC-Pro pipeline. Normalized contact maps with necessary re-formatting were then used to generate visualization files (.hic) and Pearson correlation of contact matrix using Juicer tools (version 1.5)^84^ with ‘*pre*’ command.

Eigenvector (A/B compartments) of RBLs and LCLs was estimated with 100 kb bin contact maps using ‘*pca*’ function implemented inside R mixOmics package^85^. Eigenvector was then smoothed by a moving average with a five-bin window. To correctly identify A from B compartments, Pearson correlation was calculated between Eigenvector and H3K4Me3 ChIP-seq data signals downloaded from ENCODE project (Supplementary Table 1, https://www.encodeproject.org/). For each chromosome, positive correlation resulted no changes of Eigenvector while negative correlation resulted sign flipping of the whole Eigenvector.

Continuous putative contact domain boundaries in RBLs and LCLs were identified using insulation score^86^ with parameters ‘*-is 500000 -ids 200000 -im mean -bmoe 1 -nt 0.1*’ based on normalized contact matrix under 25 kb resolution. Overlaps of domain boundaries were determined using ‘*findOverlaps*’ function in R GenomicRanges package. Starting from the first boundary on every chromosome, regions between two consecutive boundaries after merging of overlapped boundaries were defined as contact domains.

Estimation of distal to local ratios (DLR) of genome-wide 25 kb bins were accepted from recently reported protocol^42^ with minor changes to avoid infinities:${DLR}=\log 2\frac{{Distal\; interactions}+1}{{Local\; interactions}+1}$. Local interactions were set as those within 3 Mb region centered by the examined bin except the bin’s self-interactions, while distal interactions are those between the bin and regions outside the 3MB region. DLR ratios reflect the relative strength of local interactions by considering distal interactions as background, thus provide robust estimation of local regulatory status. Smaller DLR indicates increased local interactions. For each gene set selected from previous studies^13,35^, DLRs surrounding transcription starting sites of genes were aggregated together to show changes between RBLs and LCLs. DLR scores are zero center normalized to avoid potential sequencing depth biases in comparison. DLR of all genes were calculated as control.

ChIP-seq and RNA-seq data in Hi-C analysis was downloaded from ENCODE project (Supplementary Table 1) or generated in our lab previously^8,16,19,21^. For ENCODE ChIP-seq, proper datasets were selected to minimize potential biases if multiple labs producing the same sequencing library^87^. ChIP-seq data for histone modification and CTCF in RBLs and LCLs was used to visualize chromatin status changes associated to Hi-C interaction changes in Fig. 1. ChIP-seq of CTCF and EBNAs proteins was used to visualize protein binding signals at Hi-C contact domain boundaries in Fig. 2. For each contact domain, if its left and right boundaries have CTCF binding sites (downloaded from ENCODE) enriched, we identified a 4 kb sub-region of these boundaries which are centered by closest CTCF binding sites to contact domains to show potential protein binding status. For those boundaries that don’t have enriched CTCF binding, we selected the closest 4 kb regions to contact domains to show protein binding. We ranked all contact domains by the sum of CTCF coverage from their left and right selected regions in RBLs and LCLs, separately. Heatmaps of ChIP-seq data (CTCF and EBNAs) at these regions were then plotted using average coverage of 100 bp windows, and ordered based on corresponding ranks of contact domains.

### ChIP-seq signal enrichment analysis

We postulated that transcription factor (TF) occupancy, as quantified by ChIP-seq reads, around the top 1000 statistically differential loops identified from H3K27ac HiChIP in EBNA3A On and Off conditional LCLs would allow us to infer potential TFs that play a role in loop formation and transcriptional activation in each state of EBNA3A. Input normalized TF ChIP-seq occupancy data was downloaded and processed for GM12878 LCLs from ENCODE. The signal of each TF was computed within +/−2kb around each of the top 1000 differential HiChIP loops from (1) EBNA3A On and (2) EBNA3A Off. A Wilcoxon test was performed to test the signal of each TF in the EBNA3A On versus Off condition, and the log2 fold-enrichment of TF signal in On/Off was plotted.

### 4C-seq

EBNA3C-HT growth, withdrawal, and formaldehyde crosslinking were performed as described above. The 4C-seq was done as previously described^88^. The CDKN2A 4C-seq library was prepared using HindIII as the first restriction enzyme, followed by DpnII. The AICDA 4C-seq library was prepared using NlaIII as the first restriction enzyme, followed by Csp6I.

### Ribonucleoprotein CRISPR deletion of CTCF motifs

sgRNAs against the CTCF motif at the AICDA loci were designed with Benchling (https://benchling.com).

Purified Cas9 protein and sgRNA were obtained from Synthego. A detailed protocol for the assembly and nucleofection of ribonucleoprotein RNP complexes was previously described^89^. In short, 2ul of 10uM sgRNA was mixed with 1ul of purified Cas9 in 1.5 ml RNase-free microcentrifuge tubes for 5 minutes at room temperature. Nucleofection of GM12878 LCLs was performed on a 4D-Nucleofector (Lonza) in SF media (Lonza), using the DN-100 program. Cells were incubated for 24–48 hours at 37 C, before dilution seeding into 96 well plates to obtain cells from single deletion clones. An eGFP vector (Lonza) was co-trans nucleofected to assess nucleofection efficiency.

### TIDE analysis for CTCF motif deletion

Genomic DNA from Cas9 RNP nucleofected cells (pooled or single-cell clones) was extracted and PCR was performed to amplify the deletion, as previously described^89^. PCR sequencing was performed (Eton Biosciences) for the PCR product from both control or CTCF motif-targeted cells. The sequencing output was parsed using the Tracking of Indels by Decomposition (TIDE) software (https://tide-calculator.nki.nl/).

### 4C-seq data analysis

4C-seq data analysis was performed as previously described^90^. In brief, a reduced hg19 genome was first constructed with a in silico digestion using HindIII or NlaIII. Illumina sequencing barcodes and primer sequences are trimmed and the resulting reads mapped onto the reduced hg19 genome with bowtie v2 (-N 0 −5 0)^91^. Self-ligated and undigested fragments are removed, and subsequent differential 4C-seq interactions identified using the 4C-ker package^90^. The cis-interacting regions were determined by a Hidden Markov Model, using the “nearBaitAnalysis” function. Differential interactions between the EBNA3C On and Off conditions were determined using DESeq2 through the “differentialAnalysis” function with a *p*-value cutoff of 0.05.

### ChIP-seq and CUT&RUN data processing

Sequencing reads for H3K27Ac, CTCF and Rad21 in EBNA3A on and off conditions were aligned to human genome hg19 using Bowtie v2 (ChIP-seq: default settings except the parameter −k was set to 1; CUT&RUN: -I 10 -X 700 --local --very-sensitive-local --no-discordant --no-mixed --no-unal --phred33 -k 1). Uniquely aligned reads were merged from replicate samples and filtered to remove those located in blacklist regions. Peaks were called using MACS v2.2.7^92^ with default settings and parameter --SPMR to generate sequencing coverage tracks.

All GM12878 ChIP-seq data sets were downloaded from the ENCODE project portal (https://www.encodeproject.org) and all other data were previously generated as described^13^.

### HiChIP data analysis

HiChIP reads from biological replicates were pooled and processed (together with individual replicates) with the HiC-Pro pipeline (version v3.1.0) against a MboI digested hg19 genome build. Long-ranged interactions were identified with hichipper v0.7.3 using the parameters for peaks (COMBINED, ALL). Read depth normalization for long-ranged interactions identified was performed based off the number of valid PET counts per library, as determined by HiC-Pro, for visualization purposes via the WashU Epigenome Browser^93^.

The diffloops R package was used to read in long-ranged interactions called from above for downstream filtering and analysis of loops. Loops with FDR < 0.01, width > 5 kb, and in addition did not have more than 4 PETs in one biological replicates and 0 PETs in the other, were retained. Differential loops were identified in diffloops using edgeR (*p* < 0.05). Input normalized ChIP-seq reads were identified +/− 2 kb around loop anchors to calculate TF occupancy around anchors.

### Hi-C data 3D structural inference

To illustrate the difference of LCL and RBL at a genome structure level. A 3D genome structural visualization is generated based on Hi-C data at a 100k resolution. Firstly, ICE normalized matrix and related bed file generated from HiC-Pro^83^ are converted to a bedpe file with R package HiCcompare^94^. Then miniMDS (default parameters) is used to inferring genome 3D structure based on a multidimensional scaling method^49^. Finally, the output structural files from miniMDS were converted to g3d format with g3dtools^95^ and visualized using WashU genome browser^95^.

### Reporting summary

Further information on research design is available in the Nature Portfolio Reporting Summary linked to this article.

## Supplementary information

Supplementary Information

Reporting Summary

## Data Availability

The data that support this study are available from the corresponding authors upon reasonable request. All sequencing data generated in this study (HiChIP, Hi-C, 4C-seq, CUT&RUN, ChIP-seq) have been deposited in Gene Expression Omnibus (GEO) under accession ID GSE128952. All data used in the analyses including both in-house generation and publicly downloaded are listed in the Supplementary Table 1. Source data for Figs. 4c and 7c are provided as a supplementary Source Data file. Source data are provided with this paper.

## Source data

Source Data
