# Supplementary material for: A DNA tumor virus globally reprograms host 3D genome architecture to achieve immortal growth
Source: Nat Commun. 2023 Mar 22;14:1598. doi: 10.1038/s41467-023-37347-6 (PMC10033825; doi:10.1038/s41467-023-37347-6)
Supplement: Supplementary file 1 — Supplementary Information [file 41467_2023_37347_MOESM1_ESM.pdf]

Supplementary Table 1 List of sequencing data used

Dataset	Description
GEO	
GSM6886325	RBL HiC
GSM6886326	LCL HiC
GSM1181867	GM12878 LCL HiC Replicate1
GSM1181868	GM12878 LCL HiC Replicate2
GSM3688942	EBNA3C-HT 4C-seq CDKN2A 3C On Replicate 1
GSM3688943	EBNA3C-HT 4C-seq CDKN2A 3C On Replicate 2
GSM3688944	EBNA3C-HT 4C-seq CDKN2A 3C Off Replicate 1
GSM3688945	EBNA3C-HT 4C-seq CDKN2A 3C Off Replicate 2
GSM3688946	EBNA3A-HT HiChIP H3K27ac 3A On Replicate 1
GSM3688947	EBNA3A-HT HiChIP H3K27ac 3A On Replicate 2
GSM3688948	EBNA3A-HT HiChIP H3K27ac 3A Off Replicate 1
GSM3688949	EBNA3A-HT HiChIP H3K27ac 3A Off Replicate
GSM3693033	EBNA3C-HT 4C-seq AICDA 3C On Replicate 1
GSM3693034	EBNA3C-HT 4C-seq AICDA 3C On Replicate 2
GSM3693035	EBNA3C-HT 4C-seq AICDA 3C Off Replicate 1
GSM3693036	EBNA3C-HT 4C-seq AICDA 3C Off Replicate 2
GSM4289850	EBV Infection RNA PolII HiChIP Day 0 Replicate 1
GSM4289851	EBV Infection RNA PolII HiChIP Day 0 Replicate 2
GSM4289852	EBV Infection RNA PolII HiChIP Day 28 Replicate 1
GSM4289853	EBV Infection RNA PolII HiChIP Day 28 Replicate 2
GSM5456429	EBNA3A ON H3K27AC Cut&Run Rep1
GSM5456430	EBNA3A ON H3K27AC Cut&Run Rep2
GSM5456431	EBNA3A OFF H3K27AC Cut&Run Rep1
GSM5456432	EBNA3A OFF H3K27AC Cut&Run Rep2
GSM5456433	EBNA3A ON CTCF Cut&Run Rep1
GSM5456434	EBNA3A ON CTCF Cut&Run Rep2
GSM5456435	EBNA3A OFF CTCF Cut&Run Rep1
GSM5456436	EBNA3A OFF CTCF Cut&Run Rep2
GSM5456437	EBNA3A ON RAD21 ChIP-seq Rep1
GSM5456438	EBNA3A ON RAD21 ChIP-seq Rep2
GSM5456439	EBNA3A OFF RAD21 ChIP-seq Rep1
GSM5456440	EBNA3A OFF RAD21 ChIP-seq Rep2
GSM5456441	EBNA3A ON ChIP-seq INPUT
GSM5456442	EBNA3A OFF ChIP-seq INPUT
GSE29498	IB4 LCL EBNA2 ChIP-seq
GSM1197603	IB4 LCL EBNALP ChIP-seq
GSM1273052	HA EBNA3C LCL EBNA3C ChIP-seq
GSM1429820	HA EBNA3A LCL EBNA3A ChIP-seq
ENCODE	
ENCFF075VCO.bigWig	RBL H3K27me3 ChIP-seq
ENCFF167NBF.bigWig	GM12878 LCL H3K27me3 ChIP-seq
ENCFF340RFN.bigWig	RBL CTCF ChIP-seq
ENCFF364OXN.bigWig	GM12878 LCL CTCF ChIP-seq
ENCFF567EGK.bigWig	GM12878 LCL RAD21 ChIP-seq
ENCFF235BXX.bigWig	GM12878 LCL SMC3 ChIP-seq
ENCFF413PZT.bigWig	GM12878 LCL BATF ChIP-seq
ENCFF291ILI.bigWig	GM12878 LCL IRF4 ChIP-seq
ENCFF049DFX.bigWig	GM12878 LCL BCL11A ChIP-seq

Supplementary Table 2. Primers used in the manuscript.

Name	Sequence + Illumina Adapter	Barcode	Samples
AIDctcf_1F_ATCACG	AATGATACGGCGACCACCGAACACTCTTTCCCTACACG ACGCTCTTCCGATCT CGTGAT CAGCACACAGTCAAGCCATG	ATCACG	C19_1+
AIDctcf_Peak5_1F_CGATGT	AATGATACGGCGACCACCGAACACTCTTTCCCTACACG ACGCTCTTCCGATCT ACATCG CAGCACACAGTCAAGCCATG	CGATGT	C19_1-
AIDctcf_Peak5_1F_TTAGGC	AATGATACGGCGACCACCGAACACTCTTTCCCTACACG ACGCTCTTCCGATCT GCCTAA CAGCACACAGTCAAGCCATG	TTAGGC	C19_2+
AIDctcf_Peak5_1F_TGACCA	AATGATACGGCGACCACCGAACACTCTTTCCCTACACG ACGCTCTTCCGATCT TGGTCA CAGCACACAGTCAAGCCATG	TGACCA	C19_2-
AIDctcf_Illum_R	CAAGCAGAAGACGGCATACGA AAGAATTTTACCATTTCCCC		

qRT-AICDA F1 AGCGGACATTTTTGAATTGG

qRT-AICDA R1 CAGGGAGGCAAGAAGACACT

qRT_B-actin_F AAGGCCAACCGCGAGAAG

qRT_B-actin_R ACAGCCTGGATAGCAACGTACA

Fig.S1

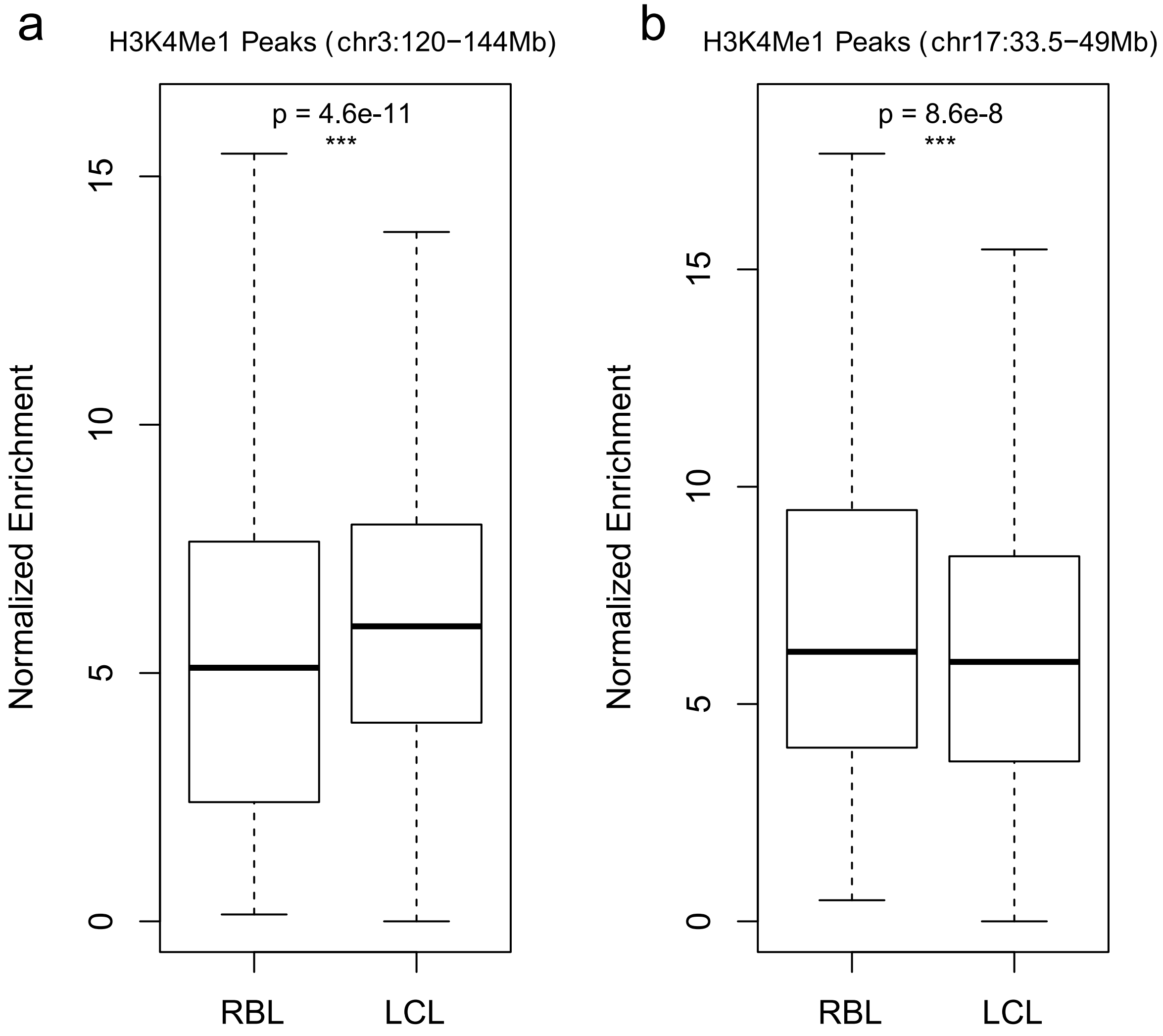

Fig. S1. Comparison of H3K4Me1 peak enrichments between RBL and LCL in the highlighted genomic blocks. a. RBL and LCL median H3K4me1 ChIP-seq signals at chr3:120−144Mb. b. RBL and LCL median H3K4me1 ChIP-seq signals at chr17:33.5−49Mb. Union peak regions were calculated between RBL and LCL. Maximum enrichments in all peaks were firstly extracted. Medians of genome-wide enrichment were then normalized between RBL and LCL to account for sequencing depth differences. P values were calcu-lated using the Wilcoxon signed rank test. Boxplot plots: center value is the medium; upper and lower bounds of boxes are upper and lower quartile, respectively; whiskers extend by 1.5*(upper quartile - lower quartile).

Fig. S2

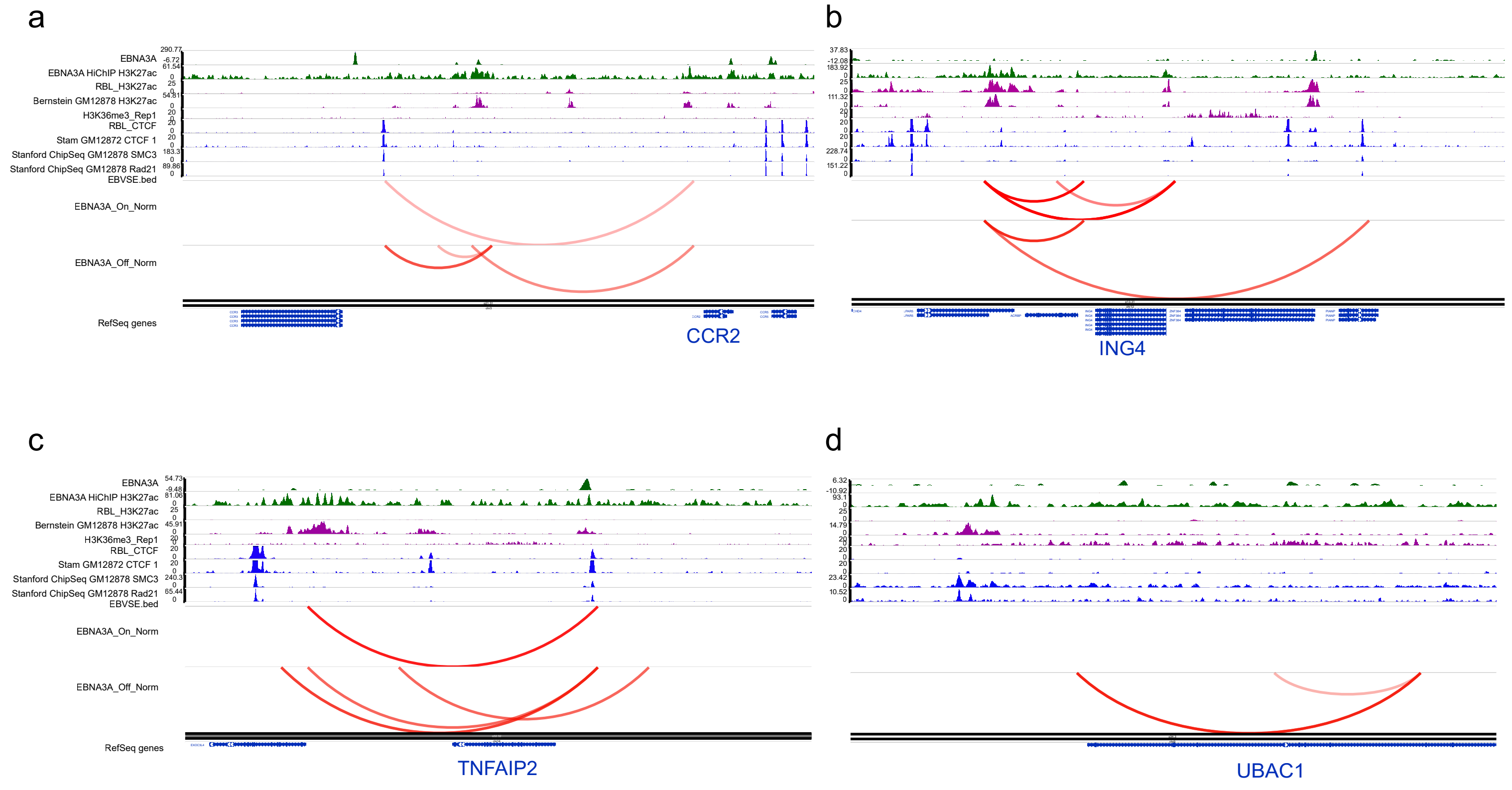

Fig. S2. Loops gained following EBNA3A inactivation. H3K27ac HiChIP loops in EBNA3A on or off conditions at a. CCR2, b. ING4, c. TNFAIP2, and d. UBAC1 loci. Loops are indicated in red lines. ChIP-seq tracks for EBNA3A, RBL and LCL H3K27ac, LCL H3K36me1, RBL CTCF, LCL CTCF, SMC3, and RAD21 are shown on top of the loops.

Fig. S3

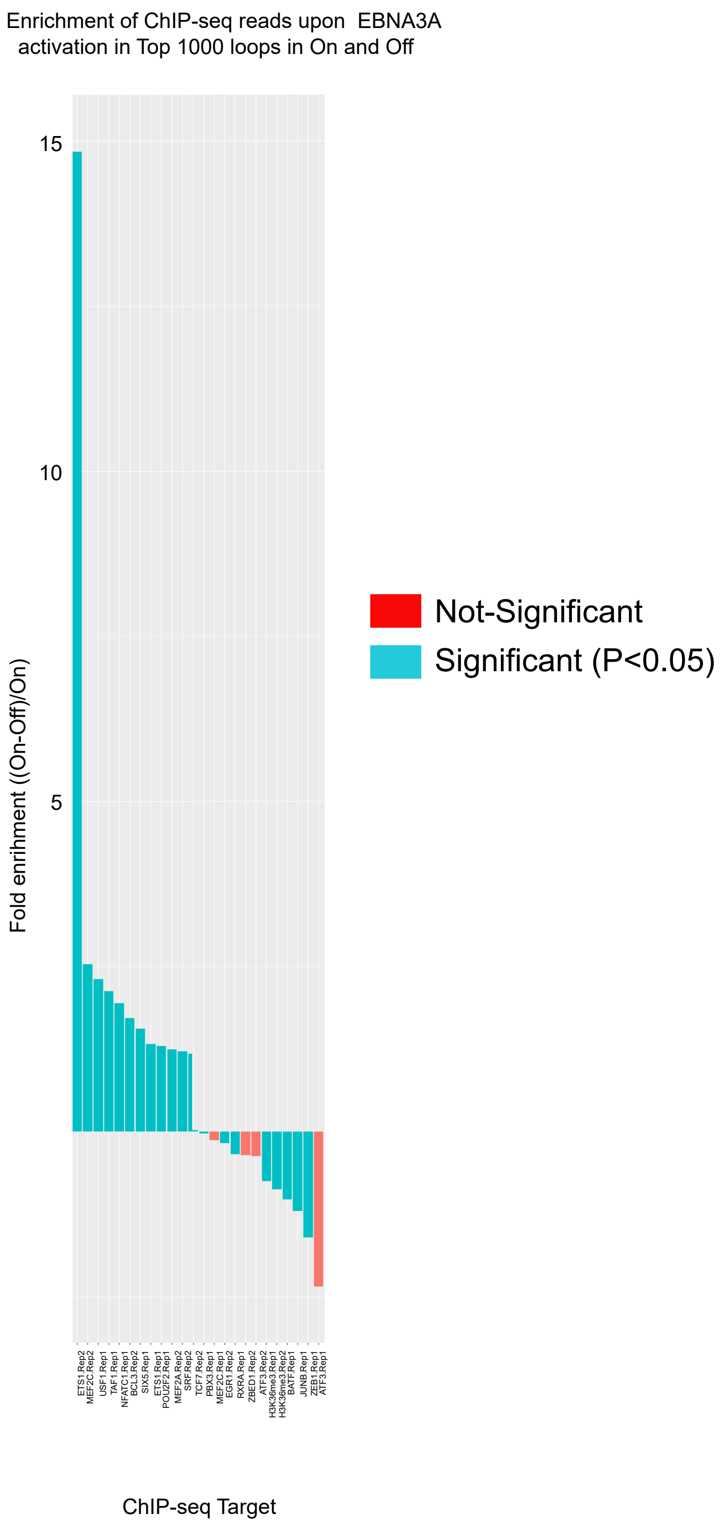

Fig. S3. TFs enriched at the loops gained or lost upon EBNA3A inactivation. ENCODE ChIP-seq data were analyzed for their signals at the sites lost or gained loops upon EBNA3A inactivation. The most significantly enriched one were shown. Orange and Teal colors represent not significant or significant interactions. Two-sided Wilcoxon rank sum test ($P < 0.05$).
