# Supplementary material for: A DNA tumor virus globally reprograms host 3D genome architecture to achieve immortal growth
Source: Nat Commun. 2023 Mar 22;14:1598. doi: 10.1038/s41467-023-37347-6 (PMC10033825; doi:10.1038/s41467-023-37347-6)
Supplement: Supplementary file 2 — Reporting Summary [file 41467_2023_37347_MOESM2_ESM.pdf]

nature portfolio

Corresponding author(s): Bo Zhao

Last updated by author(s): 02-07-2023

Reporting Summary

Nature Portfolio wishes to improve the reproducibility of the work that we publish. This form provides structure for consistency and transparency in reporting. For further information on Nature Portfolio policies, see our Editorial Policies and the Editorial Policy Checklist.

Statistics

For all statistical analyses, confirm that the following items are present in the figure legend, table legend, main text, or Methods section.

n/a	Confirmed	
☐	☒	The exact sample size (*n*) for each experimental group/condition, given as a discrete number and unit of measurement
☐	☒	A statement on whether measurements were taken from distinct samples or whether the same sample was measured repeatedly
☐	☒	The statistical test(s) used AND whether they are one- or two-sided<br>*Only common tests should be described solely by name; describe more complex techniques in the Methods section.*
☒	☐	A description of all covariates tested
☒	☐	A description of any assumptions or corrections, such as tests of normality and adjustment for multiple comparisons
☐	☒	A full description of the statistical parameters including central tendency (e.g. means) or other basic estimates (e.g. regression coefficient) AND variation (e.g. standard deviation) or associated estimates of uncertainty (e.g. confidence intervals)
☐	☒	For null hypothesis testing, the test statistic (e.g. *F*, *t*, *r*) with confidence intervals, effect sizes, degrees of freedom and *P* value noted<br>*Give P values as exact values whenever suitable.*
☒	☐	For Bayesian analysis, information on the choice of priors and Markov chain Monte Carlo settings
☒	☐	For hierarchical and complex designs, identification of the appropriate level for tests and full reporting of outcomes
☐	☒	Estimates of effect sizes (e.g. Cohen's *d*, Pearson's *r*), indicating how they were calculated

Our web collection on statistics for biologists contains articles on many of the points above.

Software and code

Policy information about availability of computer code

Data collection	No software was used for data collection. Public data was directly downloaded from ENCODE data portal.<br><br>Customized data analysis code is here: https://github.com/tenglab/EBV_HiC_manuscript/
Data analysis	In-house data analysis code was documented here: https://github.com/tenglab/EBV_HiC_manuscript<br><br>Used software/tools are listed below:<br><br>HiC-Pro V3.1.0<br><br>Juicer V1.5<br><br>Juicebox V1.11.08<br><br>mixOmics V6.20.0<br><br>matrix2insulation V1.0.0<br><br>TIDE V3.3.0<br><br>Bowtie V2.2.3

4C-ker V0.90

DESeq2 v1.22.0

MACS v2.2.7

hichipper v0.7.3

diffloops v1.17.0

HiCcompare V1.18.0

miniMDS V1.0.0

g3dtools V1.0.0

For manuscripts utilizing custom algorithms or software that are central to the research but not yet described in published literature, software must be made available to editors and reviewers. We strongly encourage code deposition in a community repository (e.g. GitHub). See the Nature Portfolio guidelines for submitting code & software for further information.

Data

Policy information about availability of data

All manuscripts must include a data availability statement. This statement should provide the following information, where applicable:

- Accession codes, unique identifiers, or web links for publicly available datasets
- A description of any restrictions on data availability
- For clinical datasets or third party data, please ensure that the statement adheres to our policy

Our sequencing data from HiChIP, Hi-C and 4C-seq have been deposited in the Gene Expression Omnibus and the accession number is GSE128952, and the reviewer token is: atihmqokpdcddir. H3K27me3 rbl Bradley Bernstein, Broad ENCFF075VCO.bigWig
H3K27me3 gm12878 Bradley Bernstein, Broad ENCFF167NBF.bigWig
CTCF rbl Bradley Bernstein, Broad ENCFF340RFN.bigWig
CTCF gm12878 Bradley Bernstein, Broad ENCFF364OXN.bigWig
EBNA2 gm12878 Bo Zhao EBNA2.bw
EBNA3A gm12878 Bo Zhao EBNA3A.bw
EBNA3C gm12878 Bo Zhao EBNA3C.bw
EBNALP gm12878 Bo Zhao EBNALP.bw
RAD21 gm12878 Richard Myers, HAIB ENCFF567EGK.bigWig
SMC3 gm12878 Michael Snyder, Stanford ENCFF235BXX.bigWig
BATF gm12878 Richard Myers, HAIB ENCFF413PZT.bigWig
IRF4 gm12878 Richard Myers, HAIB ENCFF291ILI.bigWig
BCL11A gm12878 Richard Myers, HAIB ENCFF049DFX.bigWig
MEF2A gm12878 Richard Myers, HAIB ENCFF975FYL.bigWig
GSM6886325 RBL HiC
GSM6886326 LCL HiC
GSM3688942 EBNA3C-HT 4C-seq CDKN2A 3C On Replicate 1 Dec 31, 2021 approved None
GSM3688943 EBNA3C-HT 4C-seq CDKN2A 3C On Replicate 2 Dec 31, 2021 approved None
GSM3688944 EBNA3C-HT 4C-seq CDKN2A 3C Off Replicate 1 Dec 31, 2021 approved None
GSM3688945 EBNA3C-HT 4C-seq CDKN2A 3C Off Replicate 2 Dec 31, 2021 approved None
GSM3688946 EBNA3A-HT HiChIP H3K27ac 3A On Replicate 1 Dec 31, 2021 approved None
GSM3688947 EBNA3A-HT HiChIP H3K27ac 3A On Replicate 2 Dec 31, 2021 approved None
GSM3688948 EBNA3A-HT HiChIP H3K27ac 3A Off Replicate 1 Dec 31, 2021 approved None
GSM3688949 EBNA3A-HT HiChIP H3K27ac 3A Off Replicate 2 Dec 31, 2021 approved None
GSM3693033 EBNA3C-HT 4C-seq AICDA 3C On Replicate 1 Dec 31, 2021 approved BEDGRAPH
GSM3693034 EBNA3C-HT 4C-seq AICDA 3C On Replicate 2 Dec 31, 2021 approved BEDGRAPH
GSM3693035 EBNA3C-HT 4C-seq AICDA 3C Off Replicate 1 Dec 31, 2021 approved BEDGRAPH
GSM3693036 EBNA3C-HT 4C-seq AICDA 3C Off Replicate 2 Dec 31, 2021 approved BEDGRAPH
GSM4289850 EBV Infection RNA PolII HiChIP Day 0 Replicate 1 Dec 31, 2021 approved TXT
GSM4289851 EBV Infection RNA PolII HiChIP Day 0 Replicate 2 Dec 31, 2021 approved TXT
GSM4289852 EBV Infection RNA PolII HiChIP Day 28 Replicate 1 Dec 31, 2021 approved TXT
GSM4289853 EBV Infection RNA PolII HiChIP Day 28 Replicate 2 Dec 31, 2021 approved TXT
GSM5456429 EBNA3A ON Cut&Run K27AC Rep1 Dec 31, 2021 approved NARROWPEAK
GSM5456430 EBNA3A ON Cut&Run K27AC Rep2 Dec 31, 2021 approved None
GSM5456431 EBNA3A OFF Cut&Run K27AC Rep1 Dec 31, 2021 approved NARROWPEAK
GSM5456432 EBNA3A OFF Cut&Run K27AC Rep2 Dec 31, 2021 approved None
GSM5456433 EBNA3A ON Cut&Run CTCF Rep1 Dec 31, 2021 approved NARROWPEAK
GSM5456434 EBNA3A ON Cut&Run CTCF Rep2 Dec 31, 2021 approved None
GSM5456435 EBNA3A OFF Cut&Run CTCF Rep1 Dec 31, 2021 approved NARROWPEAK
GSM5456436 EBNA3A OFF Cut&Run CTCF Rep2 Dec 31, 2021 approved None
GSM5456437 EBNA3A 4HT ChIP-seq RAD21 Rep1 Dec 31, 2021 approved NARROWPEAK
GSM5456438 EBNA3A 4HT ChIP-seq RAD21 Rep2 Dec 31, 2021 approved None

GSM5456439 EBNA3A OFFChIP-seq RAD21 Rep1 Dec 31, 2021 approved NARROWPEAK
GSM5456440 EBNA3A OFFChIP-seq RAD21 Rep2 Dec 31, 2021 approved None
GSM5456441 EBNA3A 4HT ChIP-seq INPUT Dec 31, 2021 approved None
GSM5456442 EBNA3A OFFChIP-seq INPUT Dec 31, 2021 approved None

Human research participants

Policy information about studies involving human research participants and Sex and Gender in Research.

Reporting on sex and gender	N/A
Population characteristics	N/A
Recruitment	Random
Ethics oversight	IRB approved

Note that full information on the approval of the study protocol must also be provided in the manuscript.

Field-specific reporting

Please select the one below that is the best fit for your research. If you are not sure, read the appropriate sections before making your selection.

☒ Life sciences ☐ Behavioural & social sciences ☐ Ecological, evolutionary & environmental sciences

For a reference copy of the document with all sections, see nature.com/documents/nr-reporting-summary-flat.pdf

Life sciences study design

All studies must disclose on these points even when the disclosure is negative.

Sample size	For Figure 5b, n is the number of genome-wide differential loops after altering EBNA3A on or off. It is not about sample size.<br><br>For Figure 7C, we have calculated that: a sample size of 5 in each group will have 80% power to detect a probability of 0.95 that an observation in Group 1 is less than an observation in Group 2 using a Wilcoxon rank-sum test with a 0.05 one-sided significance level.
Data exclusions	No data were excluded from the analysis
Replication	All the experiments were performed at least two-biological replicates, All attempts at replication were successful.
Randomization	N/A
Blinding	N/A

Reporting for specific materials, systems and methods

We require information from authors about some types of materials, experimental systems and methods used in many studies. Here, indicate whether each material, system or method listed is relevant to your study. If you are not sure if a list item applies to your research, read the appropriate section before selecting a response.

Materials & experimental systems

n/a	Involved in the study	
☐	☒	Antibodies
☐	☒	Eukaryotic cell lines
☒	☐	Palaeontology and archaeology
☒	☐	Animals and other organisms
☒	☐	Clinical data
☒	☐	Dual use research of concern

Methods

n/a	Involved in the study	
☒	☐	ChIP-seq
☒	☐	Flow cytometry
☒	☐	MRI-based neuroimaging

Antibodies

Antibodies used	Anti-CTCF antibody, Abcam,Catalog: ab70303<br>Anti-H3K27Ac antibody, Abeam, Catalog: ab4729<br>Anti-Rad21 antibody, Abeam , Catalog: ab992<br>Anti-RNA Polymerase II RPB1, Biolegend, Catalog: 664906
Validation	For those commercial antibodies, the validation statements are provided on the manufacturer's websites, and also the data provided in this manuscript.

Eukaryotic cell lines

Policy information about cell lines and Sex and Gender in Research

Cell line source(s)	GM12878(Coriell)
Authentication	The cell line was purchased from Coriell.
Mycoplasma contamination	No mycoplasma was detected throughout the research period.
Commonly misidentified lines<br>(See ICLAC register)	No
